# Autophagic checks and balances of cellular immune responses

**DOI:** 10.1080/27694127.2022.2058677

**Published:** 2022-03-31

**Authors:** Sukanya Chakravarty, Shumin Fan, Ritu Chakravarti, Saurabh Chattopadhyay

**Affiliations:** a Medical Microbiology and Immunology; b Physiology and Pharmacology, University of Toledo College of Medicine and Life Sciences, Toledo, Ohio, USA

**Keywords:** Alcoholic liver diseases, antiviral, auranofin, autophagy, degradation, innate response, interferon, IRF3

## Abstract

IRF3 (interferon regulatory factor 3) is a critical component of the antiviral innate immune response. IRF3 deficiency causes detrimental effects to the host during virus infection. Dysregulation of IRF3 functions is associated with viral, inflammatory, and hepatic diseases. Both transcriptional and pro-apoptotic activities of IRF3 are involved in the exacerbated inflammation and apoptosis in liver injury induced by ethanol and high-fat diets. Therefore, regulation of IRF3 activities has consequences, and it is a potential therapeutic target for infectious and inflammatory diseases. We recently revealed that IRF3 is degraded by a small molecule, auranofin, by activating the cellular macroautophagy/autophagy pathway. Autophagy is a catabolic pathway that contributes to cellular homeostasis and antiviral host defense. Degradation of IRF3 by autophagy may be a novel strategy used by the viruses to their benefit. In addition, IRF3 functions are harmful in other diseases, including liver injury and bacterial infection. A better understanding of the role of autophagy in regulating IRF3 functions has significant implications in developing therapeutic strategies. Therefore, autophagy provides checks and balances in the innate immune response.

Autophagy is an intracellular ‘self-eating’ mechanism to recycle cellular materials and yield energy during stress conditions, e.g., nutrient deprivation, DNA or organelle damage, and microbial infection. Autophagy is initiated by forming the membranous phagophore to sequester the cellular components targeted for recycling; the phagophore expands and matures to generate a double-membrane autophagosome. The autophagosome fuses with the lysosome, which contains hydrolases, to degrade the sequestered materials. The autophagic degradation products are then transported back to the cytosol for subsequent utilization. Autophagy, operating in healthy cells, contributes to cellular homeostasis. Intracellular pathogens can also activate autophagy; virus-induced autophagy can regulate viral replication and innate antiviral responses. Primarily considered an antiviral mechanism, autophagy can cause the degradation of viral proteins, host proteins used by the viruses, and virus particles by sequestering them within autophagosomes. BECN1/beclin-1, a cellular autophagy-promoting protein, causes increased autophagy to inhibit Sindbis virus/SINV replication in neurons. SHISA5/SCOTIN, an interferon (IFN)-inducible protein, recruits the hepatitis C virus (HCV) nonstructural protein NS5A into phagophores for degradation. Autophagic degradation of the picornavirus RNA genome is a cellular restriction mechanism. Viruses have also evolved strategies to evade autophagy or use it to their benefit. Herpes simplex virus (HSV-1) viral protein ICP34.5 inhibits autophagy by directly binding to BECN1. Activation of the MTOR pathway by HIV-1 in dendritic cells or vGPCR protein of KSHV are mechanisms of viral inhibition of autophagy. Viruses can also outsmart the cellular machinery by using autophagy to facilitate their replication. Picornaviruses can use autophagosomes as membrane scaffolds for RNA assembly and replication. HCV utilizes a similar mechanism by accumulating autophagosomes as sites of RNA replication. Zika virus/ZIKV induces LC3-II formation, whereas dengue virus/DENV activates the proliferation of LC3-containing membranes to their advantage. Sendai virus/SeV, respiratory syncytial virus/RSV, and HSV-1 activate AMPK, an autophagy-initiating kinase, in both autophagy-dependent and -independent ways to promote their replication.

The host-response to viral infection, via a type-I IFN response, the first line of antiviral defense, requires IRF3 activities. The pattern recognition receptors, e.g., DDX58/RIG-I and CGAS, sense the viral nucleic acids and transcriptionally activate IRF3, which translocates from the cytosol to the nucleus for inducing IFNB/IFNβ as well as other IFN-stimulated genes (ISGs) to mount a rapid cellular antiviral response. Our studies revealed a transcription-independent function of IRF3 by directly triggering the cellular apoptotic pathway RLR-induced IRF3-mediated pathway of apoptosis (RIPA) to clear the virus-infected cells. IRF3 gets activated differentially in RIPA and translocates to the mitochondria, activating the intrinsic apoptotic pathway. Both transcriptional and non-transcriptional pathways of IRF3 contribute to the optimal antiviral state of the host. IRF3, in addition to virus infection, can also be activated by bacterial infections such as *Listeria, Mycobacterium, or Neisseria* to promote bacterial pathogenesis. *Legionella* and *Salmonella* infections, conversely, activate IRF3 to benefit the host. IRF3 also plays a protective role in anti-cancer immune responses. IRF3’s role in alcohol-induced fatty liver diseases has been studied extensively; the non-transcriptional pro-apoptotic activity of IRF3 plays a detrimental role by killing hepatic cells.

We and others have recently shown that IRF3 protein can undergo autophagic degradation (Figure 1). Regulation of the immune response helps prevent the harmful effects, e.g., inflammation and cytokine storms. An unregulated IFN response can be attenuated by the cargo receptor CALCOCO2/NDP52, which recruits IRF3 to undergo autophagy-mediated degradation, whereas the deubiquitinase PSMD14 controls the autophagic degradation of IRF3 to maintain the type-I IFN response in the virus-infected cells. We uncovered that IRF3 is degraded by auranofin, an FDA-approved compound used to treat rheumatoid arthritis [1]. Auranofin activates the autophagy pathway to trigger the degradation of IRF3 but not related proteins, e.g., NFKB/NF-κB, STAT1, and MAPK14/p38. Auranofin-induced autophagic degradation of IRF3 results in the inhibition of its pro-apoptotic and transcriptional activities. Viruses might use autophagy to degrade IRF3 and inhibit its functional branches to evade the cellular antiviral responses. Other proteins in the innate immune signaling pathways are also degraded by the autophagy pathway, thereby providing additional means for the viruses to efficiently evade the antiviral responses. IRF3 activation during an alcohol-mediated hepatic injury results in pro-apoptotic activity and death of hepatic cells, eventually resulting in liver damage and cirrhosis. Therefore, in the context of alcohol-mediated hepatic steatosis, the autophagic degradation of IRF3 can be beneficial. The mouse models of alcoholic hepatitis will help reveal whether auranofin can prevent liver injury. The pharmaceutical inducers of autophagy, e.g., rapamycin and AICAR, might be used in the disease conditions where IRF3 activation can be detrimental to the host. We also speculate that auranofin might target viral proteins for autophagic degradation. In such a scenario, auranofin may function as an antiviral drug. However, whether auranofin would preferentially target IRF3 over the viral protein remains to be seen; the answer will determine whether the host or the virus wins in this context. A recent study revealed that auranofin inhibits SARS-CoV-2 replication. Does auranofin cause degradation a SARS-CoV-2 viral protein? This is an interesting question. Our study is an excellent start to addressing many exciting questions and possibilities requiring thorough investigation.

**Figure 1.**
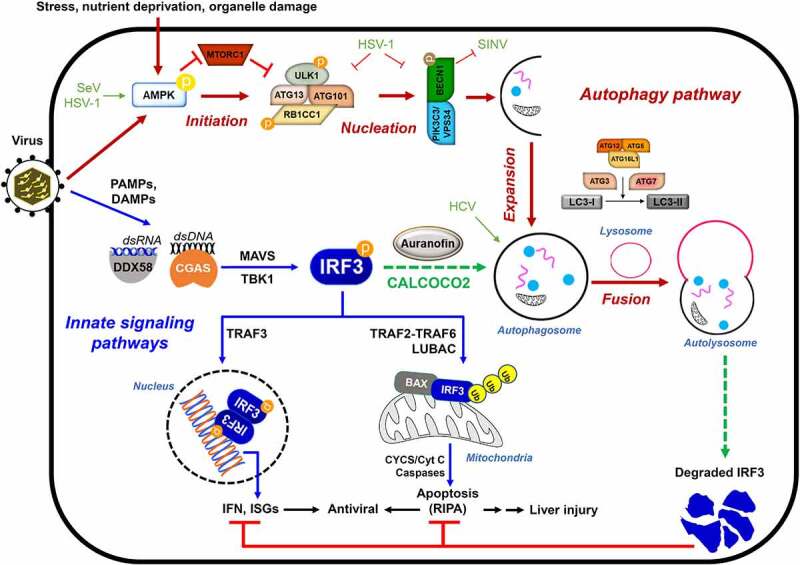
Autophagy regulates host innate immune responses. Stress conditions, including viral infection, can activate AMPK to initiate the autophagy pathway. Virus infection activates IRF3 in the transcriptional (IFN, ISGs) and non-transcriptional (apoptosis, RIPA) pathways. Autophagy can interact with the viruses directly by helping or blocking their replication; some examples (e.g., Sendai virus, HSV-1, HCV, Sindbis virus) are shown. Autophagy can also trigger the degradation of many innate signaling proteins, including IRF3, to inhibit the innate immune response. Autophagic degradation of IRF3 inhibits the transcriptional and pro-apoptotic activities of IRF3 [1].
